# Methods to estimate access to care and the effect of interventions on the outcomes of congenital disorders

**DOI:** 10.1007/s12687-018-0359-3

**Published:** 2018-03-17

**Authors:** Hannah Blencowe, Sowmiya Moorthie, Matthew W. Darlison, Stephen Gibbons, Bernadette Modell, Congenital Disorders Expert Group, A. H. Bittles, H. Blencowe, A. Christianson, S. Cousens, M. Darlison, S. Gibbons, H. Hamamy, B. Khoshnood, C. P. Howson, J. E. Lawn, P Mastroiacovo, B. Modell, S. Moorthie, J. K. Morris, P. A. Mossey, A. J. Neville, M. Petrou, S. Povey, J. Rankin, L. Schuler-Faccini, C. Wren, K. A. Yunis

**Affiliations:** 1 Centre for Maternal, Adolescent, Reproductive, and Child Health, London School of Hygiene & Tropical Medicine, London, UK; 2 PHG Foundation, 2 Worts Causeway, Cambridge, UK; 3 Centre for Health Informatics and Multiprofessional Education (CHIME), University College London, London, UK; 4 Department of Geography and Environment, London School of Economics, London, UK

**Keywords:** Congenital malformations, Interventions, Pregnancy outcomes, Estimation, Access to care

## Abstract

In the absence of intervention, early-onset congenital disorders lead to pregnancy loss, early death, or disability. Currently, lack of epidemiological data from many settings limits the understanding of the burden of these conditions, thus impeding health planning, policy-making, and commensurate resource allocation. The Modell Global Database of Congenital Disorders (MGDb) seeks to meet this need by combining general biological principles with observational and demographic data, to generate estimates of the burden of congenital disorders. A range of interventions along the life course can modify adverse outcomes associated with congenital disorders. Hence, access to and quality of services available for the prevention and care of congenital disorders affects both their birth prevalence and the outcomes for affected individuals. Information on this is therefore important to enable burden estimates for settings with limited observational data, but is lacking from many settings. This paper, the third in this special issue on methods used in the MGDb for estimating the global burden of congenital disorders, describes key interventions that impact on outcomes of congenital disorders and methods used to estimate their coverage where empirical data are not available.

**Electronic supplementary material:**

The online version of this article (10.1007/s12687-018-0359-3) contains supplementary material, which is available to authorized users.

## Introduction

**Fig. 1:**
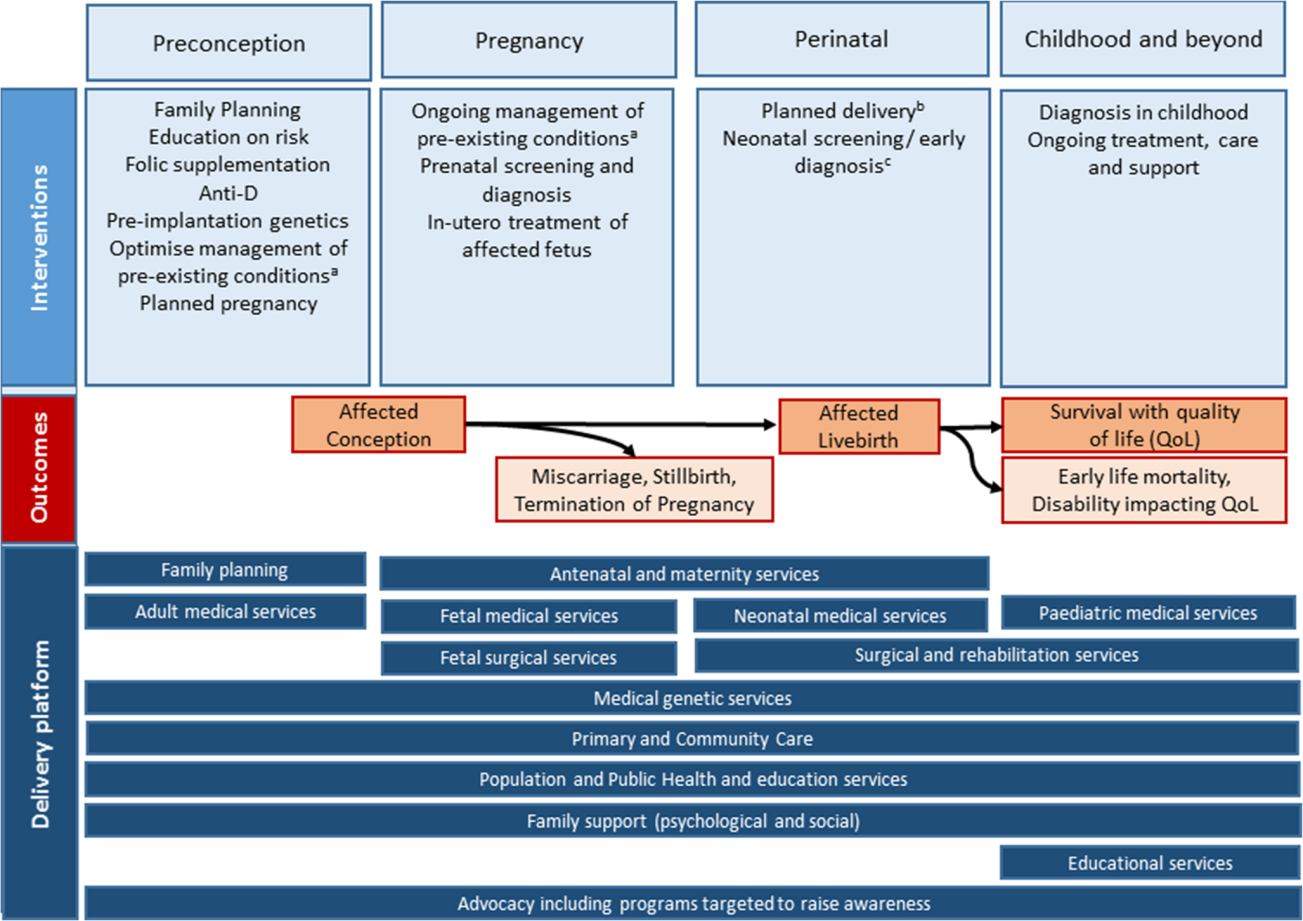
Interventions for congenital disorders along the continuum. Affected conceptions are depicted in this figure, but not quantified in MGDb. ^a^Including maximising the control and appropriate medications in pregnancy for chronic conditions including HIV, epilepsy and diabetes. ^b^Including delivery in a hospital with neonatal intensive care/surgical capabilities, planned caesarean section. ^c^Including neonatal physical exam, biochemical screening, e.g. dried blood spot, hearing screening

In the absence of intervention, early-onset congenital disorders lead to pregnancy loss, early death, or disability. A range of interventions along the life course can modify these outcomes. Preventive interventions before pregnancy include anti-D for rhesus-negative mothers following previous pregnancies to prevent iso-immunisation (Zipursky and Bhutani 2015), vitamin supplementation, e.g. folic acid food fortification or supplementation and multi-vitamin supplementation (De-Regil et al. 2015; Haider and Bhutta 2015), and pre-pregnancy counselling based on risk identification, e.g. of genetic conditions, maternal chronic conditions and infections (Hussein et al. 2015; Shannon et al. 2014; Verma and Puri 2015). Interventions during pregnancy require prenatal diagnosis. If a fetus is affected, options may include treatment during pregnancy, termination of pregnancy, or planned pregnancy, labour and neonatal care. Interventions after birth depend on early case-finding (including physical and biochemical neonatal screening), and further clinical management requiring multi-disciplinary teams with various specialist expertise (e.g. medical geneticists, paediatricians, paediatric surgeons, dieticians, physiotherapists, occupational therapists, cardiologists) and/or primary health and social care. Figure 1 shows the range of possible outcomes for affected conceptions when interventions are in place.

A range of services and delivery mechanisms are required for the provision of these interventions, which can include whole population programmes (e.g. folic acid fortification), primary health care (e.g. immunisation for rubella), more targeted clinical interventions (e.g. surgery for orofacial clefts) and social support. Furthermore, surgical and non-surgical treatments for congenital disorders can vary widely in their resource requirements, which can influence their availability in different settings, as can the perception of these disorders as an important health issue. Interventions before or during pregnancy impact on affected birth prevalence, whilst interventions after birth impact on mortality and long-term morbidity and functioning. Hence, the existence of, level of access to, and quality of interventions and services available for the prevention and care of congenital disorders will affect both their birth prevalence and the outcomes for affected individuals. Efforts to estimate the burden of these disorders should therefore take these into account (Moorthie et al. 2017c).

The *Modell Global Database* (MGDb) has been developed to seek to overcome gaps in observational epidemiological data for congenital disorders in many settings by generating estimates for these conditions combining general biological principles with available observational data (Moorthie et al. 2017c). Baseline birth prevalence (i.e. prevalence of the congenital disorder at birth in the absence of any intervention) provides a basis for making further estimates, as it provides the envelope into which all outcomes must fit (Moorthie et al. 2017c). Processes for estimating the baseline birth prevalence of specific congenital disorders within the MGDb are given in accompanying papers in this supplement (Moorthie et al. 2017a; Moorthie et al. 2017b; Moorthie et al. 2017c), with a full description available online (Modell et al. 2017). Once estimates are available for baseline birth prevalence, country-specific outcomes can be calculated based on data on the impact of specific interventions and the proportion of the population with access to these interventions. The outcomes which can be considered include birth outcomes (termination of pregnancy, fetal death, live birth); early mortality (neonatal, infant, under-5 deaths /1000 births); proportion of survivors at 5 years effectively cured, or living with mild-to-moderate or severe disability; and mean age at death.

MGDb models severe, early-onset congenital disorders that cause early death and/or life-long disability in the absence of care and present before 20 years of age. These include congenital malformations such as congenital heart disease, chromosomal disorders such as Down syndrome, and a number of inherited disorders. Full details are available in the previous paper in this series (Moorthie et al. 2017c). All these conditions have relatively constant birth prevalences in the absence of interventions. MGDb does not currently include disorders resulting primarily from exposure to external risk factors such as congenital infections, toxins or environmental factors. This is because risk varies more widely with place and time, requiring country-specific data which is currently not available.

In this article, the third in this special issue on methods for estimating the global burden of congenital disorders, we describe the interventions currently included in the MGDb and the methodology used for estimating coverage of these services (Table 1). We also describe the approach taken to estimate their impact. Provisional national and regional estimates using MGDb methodology are available online at http://discovery.ucl.ac.uk/1532179/.

**Table 1:** Included interventions affecting the birth prevalence and outcomes of congenital disorders

Timing of intervention	Intervention	Mechanism of intervention effect	Method used to estimate coverage in MGDb
Preconception	Anti-D for rhesus-negative mothers	Conversion of potential affected pregnancy to unaffected pregnancy	Modelled estimate of access to ‘optimal care’^a^
	Folic acid food fortification		Observational data or for countries with mandatory fortification and no data modelled based Wald et al. (Wald 2001)
	Identification of genetic risk, information, genetic counselling	Informed reproductive choice	Retrospective risk information coverage: modelled estimate of access to optimal care^a^ Prospective risk information coverage: for countries without data assumed to be zero coverage
Pregnancy	Identification of increased risk, information, genetic counselling. Prenatal diagnosis	Intra-uterine treatment	Not currently included
		Option of termination of pregnancy	Observational data or for countries where TOP legal prenatal diagnosis coverage estimated to be equal to optimal care* and proportion opting for TOP based on EUROCAT rates (see text for details)
After birth	Early diagnosis and care	Appropriate, timely neonatal diagnosis and care	Modelled estimate of access to optimal care^a^
		Ongoing treatment and supportive care	Modelled estimate of access to optimal care^a^

^a^Modelled estimate of access to ‘optimal care’ based on adjusted IMR (see webappendix page3)

## Estimating access to services

Information on access to services, including specialist services such as genetic counselling and paediatric surgery, including cardiac surgery, is important to estimate the burden of congenital disorders. In MGDb, information on access to individual elements or packages of services is used where available for a specific country. However, for many countries, comprehensive data on the coverage of these services are not available. For these countries, we sought to provide an estimate of access to a comprehensive package of ‘optimal care’. For the purposes of these estimates, optimal care is defined as the standard of care available in high-income settings with equitable access to services. In principle, this could be achieved using a combination of relevant health index proxies, such as average life expectancy, or neonatal, infant or under-5 mortality, or the proportion of the population that is urbanised. However, since these measures are all highly correlated (online resource: (Figure i, Table i)) and consistent with previous global perinatal estimates, we chose to select a single proxy indicator to estimate access to optimal care services.

The World Health Organization’s Child Health Epidemiology Reference Group (CHERG) used neonatal mortality rates (NMR) to define levels of access to care for estimates of long-term outcomes following neonatal conditions. This decision was based on their collective expert experience that a NMR of > 30/1000 indicates very limited access to health services, but that access increases rapidly as countries pass through the development window, and a neonatal mortality < 5/1000 indicates near 100% access (Blencowe et al. 2013). Infant mortality rate (IMR) is closely correlated with NMR (coefficient of correlation = 0.93, Online resource Figure ii). In MGDb, we use IMR, for which country estimates (1950–2015) and projections to 2100 are available (UN Population Division 2015), in preference over NMR which is available for a more limited time period, thus allowing the generation of estimates for historical time periods, and for future projections under different intervention scale-up scenarios. In addition, in many countries, sub-national data are more readily available for infant mortality than for other candidate indicators, allowing sub-national estimates to be generated.

**Fig 2:**
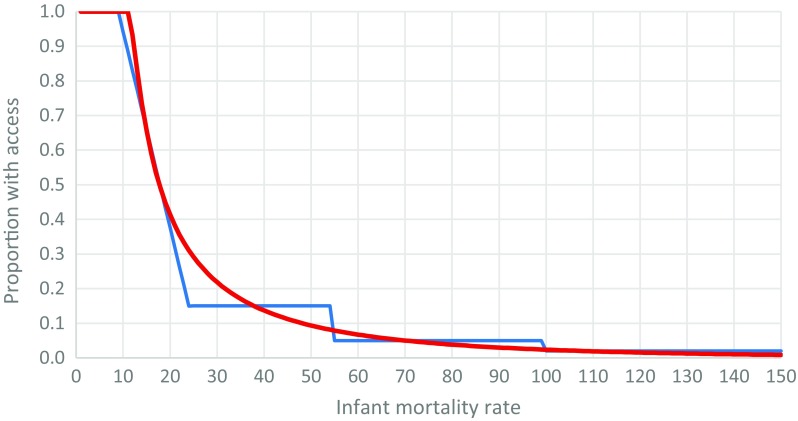
Relationship of infant mortality rate to estimated access to care. Blue line shows estimated access to care using CHERG methods (Table 2) with smoothing from NMR 5–15 (webappendix Fig. 3). Red line shows continuous curve fitted to the stepped curve used in MGD^b^

Table 2 shows the five neonatal mortality groups used by CHERG, the corresponding infant mortality groups and estimated proportion of the population with access to services (Blencowe et al. 2013). This method has the advantage of encapsulating the experience of experts within CHERG, but its step-wise nature gives rise to undesirable discontinuities, particularly as, with time, countries move across boundaries (Online resource Figure iii). We refined this method further by deriving a curve to represent the estimated relationship between access to care and IMR, based on the Beta family of distributions (see Online resource page 4 for details). Figure 2 shows the general relation of infant mortality to estimated access, calculated using the mortality groups in Table 2 (blue line) and the continuous curve that was fitted to it (red line).

**Table 2:** Estimated proportion of the population with access to services by mortality group

Group no.	Mortality level	Services for congenital disorders	Neonatal mortality range	Corresponding infant mortality range^a^	Estimated % access to optimal care^b^
**1**	Very low	Optimal	≤ 5	≤ 9	100%
**2**	Low	Evolving	6–15	10–24	50%
**3**	Moderate	For some	16–30	25–54	15%
**4**	High	For few	31–45	55–99	5%
**5**	Very high	For none	> 45	100 plus	0%

^a^Five infant mortality groups corresponding to the CHERG neonatal mortality groups were defined using the relationship between IMR and NMR in 1990 (webappendix Fig. 2)

^b^Data source: Child Health Epidemiology Reference Group (CHERG) described in Blencowe et al. (2013)

### Adjustments to estimates of access to care based on infant mortality rates

We undertook adjustments to the IMR as a proxy indicator of access to care to account for the effects on IMR of parental consanguinity and HIV infection as detailed below.

#### Adjustment of IMR for prevalence of parental consanguinity

Early-onset congenital disorders contribute significantly to infant mortality; hence, there is potential circularity in using IMR to estimate access to care. This is minimal for chromosomal disorders and congenital malformations where the baseline prevalence is similar in most populations (Moorthie et al. 2017a, b). For single gene disorders, which may be consanguinity-associated, the baseline prevalence differs substantially between populations. We therefore adjusted the IMR to account for the increased contribution of infant deaths from consanguinity-associated disorders to overall IMR to seek to improve the estimate of access to care services.

The consanguinity-adjusted IMR is calculated as follows:

$$ \mathrm{Consanguinity}\_\mathrm{adjusted}\ \mathrm{IMR}=\mathrm{IMR}-\mathrm{cIMR} $$

where cIMR is the consanguinity-associated IMR calculated from (a) local coefficients of consanguinity (a measure of gene pairs that are identical in offspring because they are inherited from recent common ancestor(s)) (Bittles 2001)); (b) mortality from consanguinity-associated disorders in the absence of care and with optimal care (Bittles and Black 2010; Bittles and Neel 1994; Bundey and Alam 1993); and (c) estimated access to care as described (see Online resource page 4). This initial adjustment over-estimates consanguinity-associated mortality and so over-reduces the infant mortality rate; hence, further iterations were undertaken until a stable adjusted IMR was achieved (see Online resource page 5). After two iterations, further iterations made little difference to the adjusted IMR, and therefore, two iterations were undertaken.

**Fig. 3:**
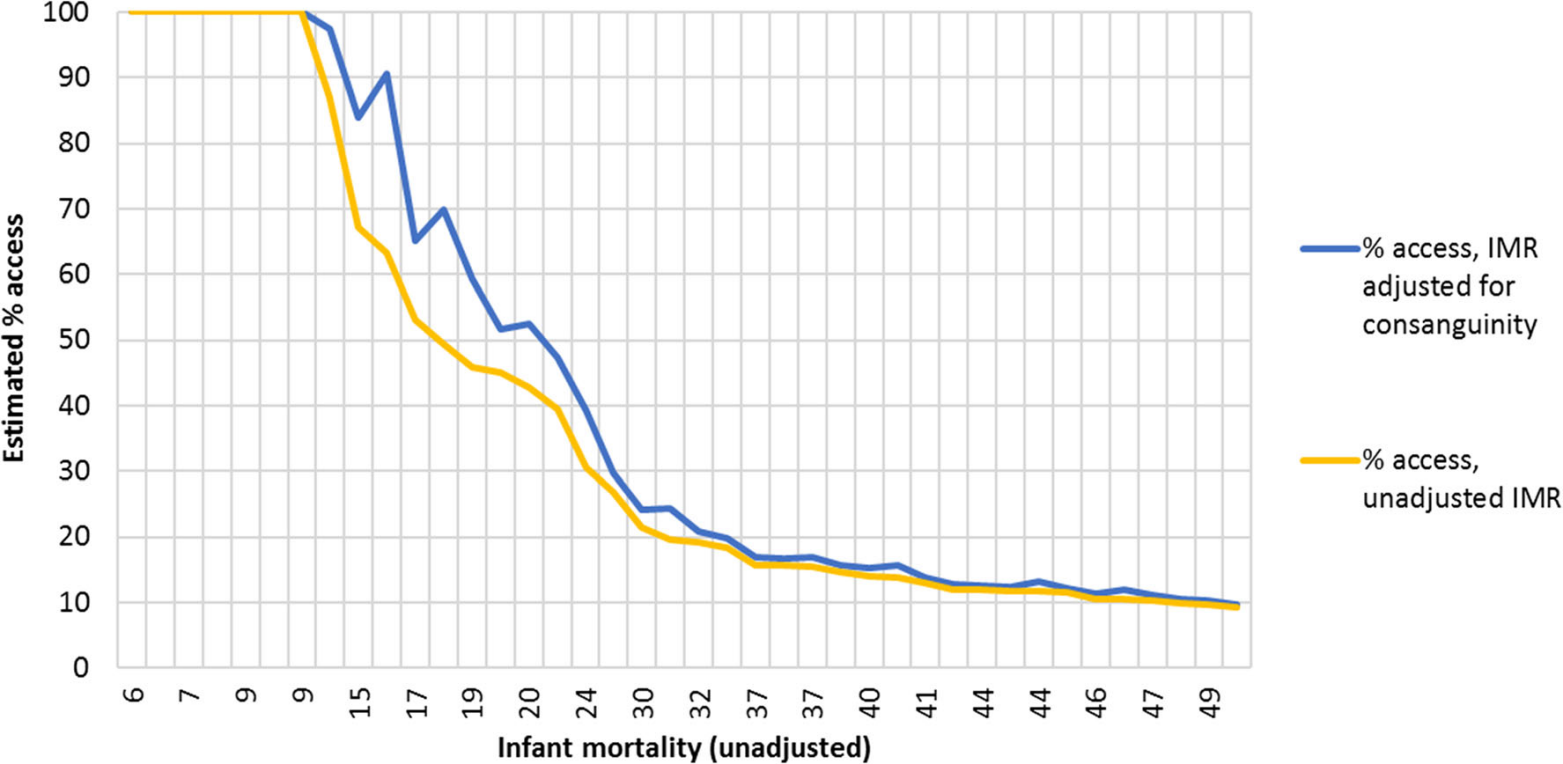
Relationship between infant mortality and estimated access to care for 40 low mortality countries. Low mortality is defined as an IMR less than 50 per 1000 live births

This adjustment has a marked effect for countries with a high prevalence of parental consanguinity that are on the steepest part of the development curve (i.e. infant mortality between 10 and 35/1000), many of which are located in the Eastern Mediterranean region (Fig. 3). The effect is minimal at low levels of IMR, where high levels of access to care limit the number of infant deaths, and at high levels of IMR where the proportion of all mortality attributable to these disorders will be relatively low.

#### Adjustment of IMR for AIDS-related infant mortality

The HIV/AIDS epidemic has had a substantial effect on infant mortality in a number of countries over the past two decades, particularly in sub-Saharan Africa (Institute for Health Metrics and Evaluation (IHME) 2015; Liu et al. 2017). In these settings, using an unadjusted IMR may underestimate the access to care, and hence, we undertook a further adjustment to the IMR by subtracting HIV-related infant mortality (hivIMR).

Although the contribution of HIV/AIDS to infant mortality in sub-Saharan Africa is substantial, it has a relatively small effect on estimated access to services in most countries in the region because infant mortality is still > 40/1000.

#### Estimates of access to care adjusted for consanguinity and HIV/AIDS

The final adjusted IMR is calculated as follows:

$$ \mathrm{Final}\_\mathrm{adjusted}\ \mathrm{IMR}=\mathrm{consanguinity}\_\mathrm{adjusted}\ \mathrm{IMR}-\mathrm{hivIMR} $$

Final access to care was estimated using the adjusted IMR and the access to care equation (Online resource page 4, Table ii). The effects of the adjustments are shown by WHO region in Table 3. Currently, in MGDb, access to care is a binary variable. We have assumed that those who do not have access to optimal care have no access to any ‘supportive medical services’. This is an over-simplification which may under-estimate the impact of interventions in some settings as services are scaled-up. In particular, in low- and middle-income settings, services requiring very high level of trained and supportive staff, for example surgical care for complex congenital heart disease, are likely to be scaled-up later than services requiring less intensive diagnostic and surgical skills, for example surgical repair of oro-facial clefts.

**Table 3:** Effects of adjustment for consanguinity and HIV/AIDS on access to care estimates by World Health Organization (WHO) region

WHO region or sub-region	Births, 1000 s	IMR (WPP)	Contribution of		% with access to optimal care, based on	
			Consanguinity-associated IMR	HIV-related IMR	Unadjusted IMR	Final adjusted IMR (% increase in access)
AFR total	34,230	62.6	1.49	1.11	7.7	8.4 (5.5)
AMR total	15,319	15.8	0.15	0.01	63	63 (0.7)
EMR total	17,323	45.6	4.35	0.05	25	30 (21.5)
EUR total	11,296	10.7	0.52	0.01	87	88 (1.8)
SEAR total	37,304	37.3	1.16	0.03	18	19 (4.0)
WPR total	24,368	13.3	0.13	0.01	85	86 (0.9)
World	139,840	35.8	1.30	0.29	39	40

*AFR* African region, *AMR* American region, *EMR* Eastern Mediterranean Region, *EUR* European region, *SEAR* Southeast Asian region, *WPR* Western Pacific region

## Interventions that impact on birth outcomes

This section provides further details on the interventions that impact on birth outcomes currently included in the MGDb (Table 1).

### Prevention of iso-immunisation of Rh-negative women

Prevention of rhesus isoimmunisation following miscarriage or delivery protects the next pregnancy from rhesus haemolytic disease of the newborn. In high-resource countries, a pincer movement of improved prevention and improved management has practically eradicated mortality and morbidity due to Rh incompatibility (Zipursky and Bhutani 2015). Routine post-partum administration of anti-D protects most Rh-negative women. The addition of routine anti-D during pregnancy has reduced maternal immunisation to almost zero (Clarke and Whitfield 1984; Tovey 1984). Whilst rhesus negativity is commonest in populations of European origin, it can occur in any population. Absence of national policies integrating diagnosis and prevention into pregnancy care in many low- and middle-income countries (LMICs) have led to it remaining an important preventable cause of adverse birth outcomes in these settings.

Previous estimates have sought to quantify the coverage of anti-D based on sales data of Rhesus immunoglobulin from registered companies (Bhutani et al. 2013). Whilst this previously provided a reasonable estimate of coverage, recent proliferation in the number of manufacturers and the increasing use of monoclonal substitutes now make this approach challenging. In MGDb, the coverage of anti-D is assumed as a minimum to be equal to the estimated access to optimal care. As this may underestimate access in countries where detection of Rh negativity and provision of anti-D is considered standard obstetric practice and coverage will depend on reach of maternity services, maximum coverage in these countries is assumed to be equal to the coverage of four antenatal care visits (see Modell et al. (2017) for further details). Country-specific data on the utilisation of anti-D are needed to improve estimates.

### Folic acid

Maternal folate intake influences the risk of neural tube defects (NTDs), including anencephaly, spina bifida and encephalocoele. Although adequate intake of folic acid does not prevent 100% of cases, due to other environmental and genetic factors that influence the risk of NTD, studies have shown it to be an effective preventative strategy (Blencowe et al. 2010). There is strong evidence to support a positive effect of mandatory folic acid fortification on NTDs (Atta et al. 2016; Williams et al. 2015; Zaganjor et al. 2016).

**Fig. 4:**
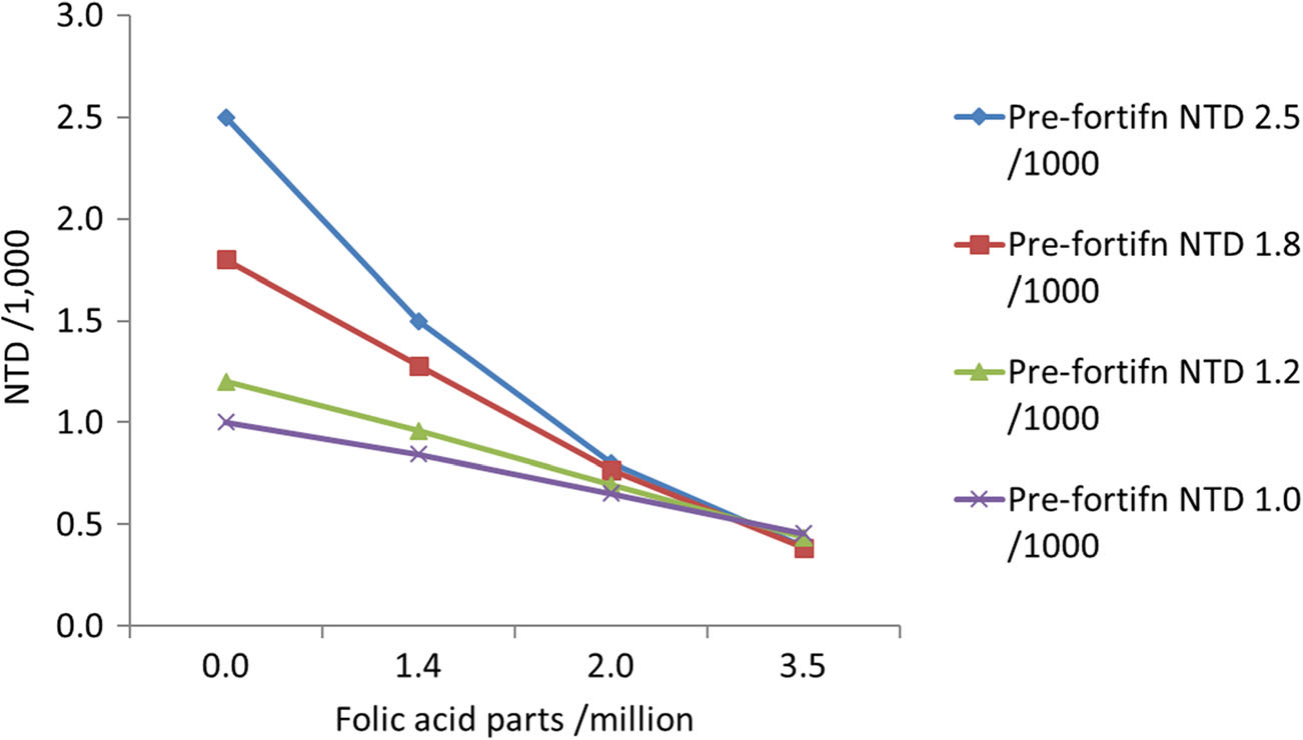
Effect of different doses of folic acid flour fortification in relation to initial birth prevalence of neural tube defects. Data source: Wald (2001). *x* parts/million = *x* μg folic acid per 100 g flour

The effect of folic acid food fortification is dependent on dose and affected birth prevalence. Small doses lead to a marked reduction when birth prevalence is high, and earlier studies showed that fortification with around 2 ppm will reduce total neural tube defect birth prevalence to < 1/1000 births, regardless of the pre-fortification birth prevalence (Taruscio et al. 2003; Wald 2001; Zimmerman 2010) (Fig. 4, Online resource Table iii). Large-scale surveillance data with prenatal ascertainment from the USA have reported a reduction of birth prevalence of spina bifida and anencephaly to around 0.7/1000 (Williams et al. 2015). Accounting for encephalocoeles, assuming their prevalence to be 11.5% of that of neural tube defects, we have assumed that this baseline rate for ‘non-folic-acid-preventable’ neural tube defects to be 0.77/1000 and that this applies to all populations (Arth et al. 2016; Williams et al. 2015) (Online resource Table iv).

In MGDb, observed pre- and post-fortification birth prevalences of NTDs have been used when available. For countries without observational data, we have estimated folate-preventable neural tube defects as the total observed (or estimated) baseline birth prevalence (in the absence of folic fortification) minus the non-folic-acid-preventable neural tube defects (0.77/1000). Evidence suggests that folic acid supplementation or voluntary fortification has little impact, and we have therefore assumed no effect of these interventions (De-Regil et al. 2010; Khoshnood et al. 2015). For countries with mandatory fortification but without observational data, we have estimated the number of NTDs prevented from the fortification level using the data in Fig. 4, and the estimated proportion of the population reached by fortification. It is assumed that folic acid food fortification has the same effect on all neural tube defects, although data from the USA suggest a more marked effect on spina bifida than on anencephaly (Alasfoor et al. 2010; Besser et al. 2007; NBDPN 2009) (Online resource Table iv).

Some studies also support a possible effect of folic acid on other malformation groups including orofacial clefts and congenital heart disease (Bedard et al. 2013; Botto et al. 2006; Feng et al. 2015; Johnson and Little 2008; Leirgul et al. 2015; Li et al. 2012; Liu et al. 2016; Wehby and Murray 2010). Whilst the evidence is not yet conclusive, it is biologically plausible, and a small effect of folate fortification on oro-facial clefts and congenital heart disease is therefore included currently in MGDb (Modell et al. 2017).

There is interest in the potential of vitamin B12 to reduce vitamin-sensitive congenital malformations, and vitamin B12 is included in the food fortification policies in some countries. However, to date, the evidence to quantify the effectiveness for this approach is lacking, and we have not estimated its effect within MGDb.

### Prenatal diagnosis and termination of pregnancy

The common objective of prenatal diagnostic services is to provide pregnant women with definitive fetal diagnoses. Definitive fetal diagnoses can facilitate informed discussions with parents around management options. Where termination of pregnancy (TOP) for fetal impairment is legal, these discussions include the option of termination where culturally acceptable and the implications of continuing with an affected pregnancy. However, in all settings, prenatal diagnosis allows women with continuing pregnancies to receive supportive care and tailored management throughout pregnancy, childbirth and into childhood. Diagnosis may be through fetal anomaly scanning or laboratory techniques to identify biomarkers that indicate an affected fetus. There are no readily accessible observational data on the spread of methods for, and utilisation of, prenatal diagnosis in most countries. Prenatal screening policies, when present, vary across countries from actively offering screening and prenatal diagnosis to every pregnant woman, to more restricted policies, e.g. covering only older women or those with a recognised increased risk. The type of screening policy impacts on pregnancy outcome, e.g. in European countries where TOP is legal, a restricted screening policy is associated with lower rates of TOP for Down syndrome and spina bifida compared to countries with universal screening (Online resource page 10) (Boyd et al. 2008).

TOP for congenital disorders is not only affected by screening policy and availability of prenatal diagnosis but also by the legal status, national policy and clinical practice of TOP for fetal impairment in the country (UN Population Division 2013). The assumptions currently used are shown in Table 4, with further details in Online Resource Table v-vii. High-quality observational data on TOP are available for 25 countries in Europe, North America and Australasia, and these are used as reported (Group A) (European Surveillance of Congenital Anomalies (EUROCAT) n.d.; International Clearing House for Birth Defects n.d.). For countries where there are no observational data, but termination for fetal impairment is legal (Group B) (United Nations 2014), it is assumed that (a) prenatal diagnosis is incorporated into routine pregnancy care as it develops; (b) for those with access to prenatal diagnosis, average EUROCAT rates for termination of pregnancy apply for all congenital anomalies except Down syndrome and spina bifida; and (c) unless there is an explicit universal screening policy, termination rates for Down syndrome and spina bifida are 50% of average EUROCAT rates.

**Table 4:** Assumptions regarding termination of pregnancy (TOP) for congenital disorders worldwide

Country group	Status of TOP	Data availability	Assumption	Evidence to support/challenge this assumption	Impact on estimate of birth outcome
A	Legal	Observational from registries	All terminations are reported and recorded	Even where registry coverage high and quality high, minimal under-reporting may occur	Minimal underestimation of number of TOPs
B	Legal	No data	Access to prenatal diagnosis can be predicted using model for ‘optimal care’ and that when diagnosed the proportion of women opting for TOP is equal to the EUROCAT average	Based on plausibility. Further evidence needed to test this assumption. Countries to be encourage to develop registry systems where possible to strengthen available data	Not known
C	Unclear	No data	No terminations take place unless documented official medical guidance, fatwas or other authoritative documents are available stating otherwise.	TOP likely available to a subset of the population in many of these countries, but supporting evidence not available.	Likely to substantially underestimate the number of TOPs, and hence overestimate the number of stillbirths and affected births
			Where evidence of widespread availability of TOP, access to prenatal diagnosis can be predicted using model for optimal care and that when diagnosed the proportion of women opting for TOP is equal to the EUROCAT average.	Pakistan evidence of acceptability of TOP especially for severe conditions (Jafri et al. 2015)	Not known
D	Illegal	No data due to legal status	No pregnancies are terminated	TOP likely available to a subset of the population in many of these countries, but supporting evidence not available.	Will underestimate the number of TOPs, and hence overestimate the number of stillbirths and affected births

For countries with no high-quality observational data where TOP for fetal impairment is illegal or where its status is unclear, we undertook consultations with experts and a Web-based review for evidence to support the practice of TOP. Evidence to suggest the widespread practice of offering the option of TOP for fetal impairment was found for seven countries (group C) Table 4, Online resource Table vi). For all other countries, we assume that no pregnancies are terminated for fetal impairment (group D) (UN Population Division 2013). A limitation of this approach is that in many countries, there are gaps between legal status, official policy and clinical practice, and this approach by underestimating the number of TOPs will effectively overestimate the number of affected births and congenital associated mortality and disability (Online resource page 11).

### Genetic counselling and associated medical genetic services

Any genetic diagnosis involves the family as well as the presenting individual. Relatives need information on the mode of inheritance and possible health and reproductive risks for themselves, access to definitive diagnosis when this is available and supportive genetic counselling.

Globally, family studies can often prospectively identify relatives at risk for dominant and X-linked disorders. However, for recessive disorders, the great majority of at-risk couples are identified *retrospectively* (i.e. through the diagnosis of the first affected child).

For some recessive conditions risk can also be identified *prospectively* (i.e. before the birth of any affected child) by systematic carrier screening, or rarely through prospective family studies, especially in populations with high rates of consanguineous marriage (Ahmed et al. 2002). Systematic carrier testing is not yet feasible for most single gene disorders and is practised on a large scale only for haemoglobin disorders, although technological advances, such as genome scanning, may change the feasibility of widespread prospective identification of a greater number of disorders in the future (Bell et al. 2011; Gelb 2013; Himes et al. 2017; Teeuw et al. 2014; Yang et al. 2013). Evidence from beta-thalassaemia pre-marital screening shows that non-directive risk information has little effect on final choice of marriage partner (Alhamdan et al. 2007; Angastiniotis and Hadjiminas 1981; Zeinalian et al. 2013), even when prenatal diagnosis is not available (Alhamdan et al. 2007; Stamatoyannopoulos 1974). At the population level, a range of factors determines the effect of risk information on affected birth rate. These include whether risk is detected retrospectively or prospectively, the reproductive aims of at-risk couples, their access to services and the population norm for final family size.

### Potential effect of retrospective risk information for recessive disorders

**Fig. 5:**
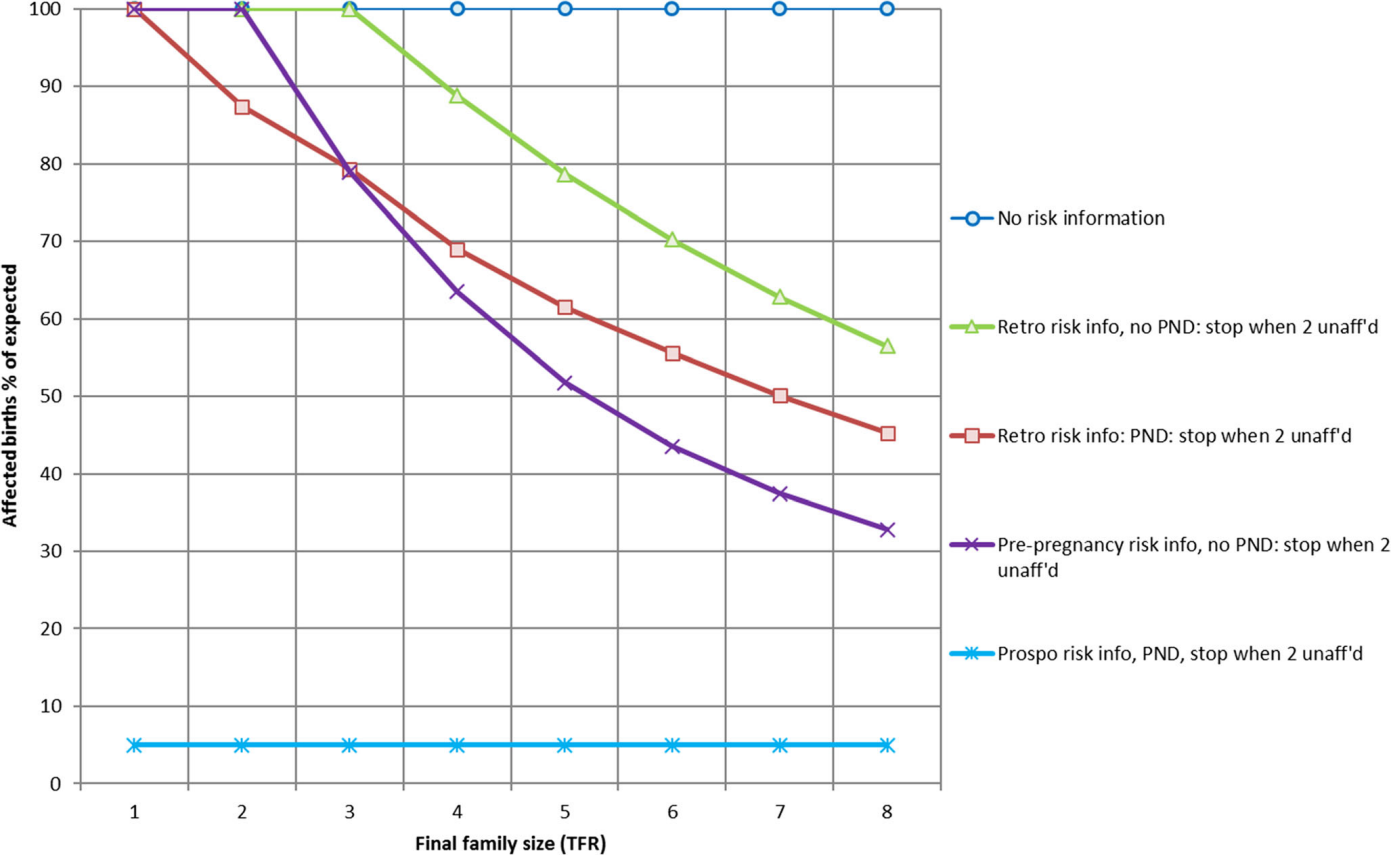
Estimated effect of genetic counselling for severe recessive disorders, in relation to family size. TFR = total fertility rate; Retro risk info = retrospective risk information; Prospo risk info = prospective risk information; PND = prenatal diagnosis; Unaff’d = unaffected

When affected children are not diagnosed, parents cannot be informed of their risk and in MGDb, they are assumed to reproduce according to the population norm, with 100% of expected birth prevalence for the condition (Fig. 5). The Hardy-Weinberg equation is used to estimate baseline affected birth prevalence (Aguzzi et al. 1978). The actual birth prevalence may be higher due to replacement of affected children who have died.

Following the diagnosis of an affected child, parents informed of the recurrence risk may take steps to avoid another affected pregnancy. When preimplantation or early pregnancy diagnosis services are available, at-risk couples can complete their desired family size while avoiding the birth of a second affected child. When prenatal diagnosis is not available, if all retrospectively detected at-risk couples stopped reproducing in order to avoid recurrence, the birth prevalence would similarly fall. However, in practice for countries where observational data are available such as the UK and Iran, the majority of at-risk couples with fewer than two healthy children undertake further pregnancies in the hope of obtaining unaffected children (Petrou et al. 2000; Safari Moradabadi et al. 2015). Data on reproductive practices from other higher-fertility settings are not available; however, the maximum theoretical possible effect of retrospective risk information at the population level is a 50% fall in affected birth prevalence for settings with an average final family size is six or more (Fig. 5). However, as average family sizes are decreasing rapidly in many settings, including LMICs, the current global average is 2.5 children. Hence, the maximum possible effect of retrospective risk information at a global level would be a 15% fall in affected births, although the effect would be greater for countries that still have high fertility (UN Population Division 2015).

### Potential effect of prospective risk information

The identification of at-risk couples before they have any affected child, for example through premarital or preconception screening, permits a wider range of options (Petrou et al. 2000). In practice, many such couples limit their family to two healthy children (Boyd et al. 2008). Figure 5 shows the theoretical maximum possible effect of prenatal diagnosis on the calculated fall in affected birth prevalence when carrier couples are detected prospectively, and stop reproducing when they have two healthy children. Where prenatal diagnosis is not available, the affected birth prevalence would fall by around 50% when the norm for average family size is six or more, but there is no effect when it is less than 3. When prenatal diagnosis is available, the effect depends on the proportion of at-risk couples who access these services, and the perceived severity of the disorder. Evidence from β-thalassaemia screening programs shows a maximum reduction in affected births of over 95% (Fig. 5) (Angastiniotis et al. 1986; Mitchell et al. 1996; Zeinalian et al. 2013); whilst evidence from sickle cell disorder screening in the UK, which is perceived as a less severe disorder, found that only 15% of at-risk couples opt for prenatal diagnosis and TOP.

In conclusion, a package of prospective detection of genetic reproductive risks, coupled with access to comprehensive family planning and prenatal diagnosis services, is currently the most effective intervention to substantially reduce the birth prevalence of inherited genetic disorders. Ideally, all women and their families should have access to this full package and sufficient information and support to make their reproductive choices, which will vary depending on many factors including the individual’s culture and beliefs. However, access to this full package of patient services remains low, even in many high-income settings, and even when resources are available is frequently dependent on political choice regarding population screening. A global network of collaborators provided information on the coverage of prospective risk screening used in MGDb (Modell and Darlison 2008). In practice, retrospective risk identification is more commonly available. In MGDb, we assume that the proportion of affected children diagnosed and whose parents received genetic counselling is equal to the proportion with access to services, calculated as above.

## Included interventions that impact on mortality and disability

The majority of individuals affected by congenital disorders require specialist management, frequently with ongoing care and support throughout life. Treatment of congenital malformations often involves surgical repair and in some cases, as with orofacial clefts, surgery can result in effective cure. However, many individuals, particularly those who have undergone complex surgery including cardiac surgery, have a residual risk of death or disability and require life-long surveillance, with intervention when appropriate. Early diagnosis, e.g. through neonatal screening programmes, can improve outcomes for affected individuals and families. It can enable early initiation of treatment including rehabilitation where available thus optimising outcomes, early supportive care for all individuals and families and can assist families with future reproductive choices.

The evolution of services over time further complicates the assessment of trends in mortality and the survival of children with congenital disorders. Table 5 summarises the evolution of these interventions by disorder group and decade. The timeline indicates introduction of the interventions, but this does not equate to their universal deployment, even in high-income settings.

**Table 5:**
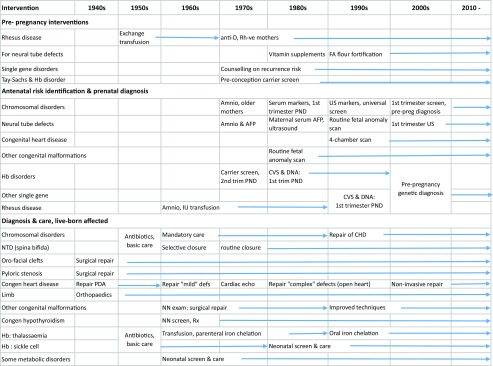
Timeline of interventions that impact on mortality and disability outcomes for congenital disorders

Full documentation of survival in the absence of care is available for many severe disorders, because it requires only a short period of observation when life expectancy is short, for example, Trisomy 13 and 18. There are considerable historical data documenting survival to 20 years at different stages in the evolution of care, e.g. the data of Czeizel and Sankaranarayan (1984) (Czeizel and Sankaranarayanan 1984). Observational data on survival up to 20 or 30 years with current standards of optimal care are available for many disorders, including congenital cardiac disorders (Tennant et al. 2010). Longer-term survival data are available for some disorders including Down, Turner and Klinefelter syndromes, oro-facial clefts and haemoglobin disorders (Baird and Sadovnick 1988; Bojesen et al. 2004; Christensen et al. 2004; Frid et al. 2004; Modell et al. 2008; Platt et al. 1994; Price et al. 1986). These and other available observational data are used to estimate the survival in the presence and absence of optimal care (further details are available online (Modell et al. 2017)). One limitation of this approach is that the care received by affected individuals has evolved over time which may affect the application of these data to more recent births.

In MGDb, early mortality, disability and cure are all calculated using estimated access to diagnostic and treatment services by the formula described above, and the effectiveness of the intervention on outcomes. The same approach applies for long-term estimates of years of life lost, lived with disability or cure, numbers of living patients and projected effects of policy change.

## Conclusion

Congenital disorders are highly diverse in their aetiology and outcomes. Their diagnosis and management therefore requires diverse interventions involving numerous different specialist clinical and genetic services. A large number of interventions, including improving prepregnancy folate status, anti-D for rhesus-negative mothers, prenatal diagnosis with the option of termination of pregnancy where culturally acceptable, or planned delivery, and early diagnosis and treatment have led to a substantial reduction in the burden of congenital disorders in high-income countries over the past 50 years. The largest burden of these disorders therefore currently lies in low- and middle-income countries. However, in the absence of strong diagnostic systems, death and disability due to congenital disorders, even when recorded, may be attributed to other causes, such as infection.

Interventions have a potential to impact on the overall number of affected conceptions, and the distribution of outcomes of these pregnancies, and hence, it is important to consider access to services when assessing overall burden. In the absence of robust observational data, estimates generated using the MGDb methodology can be used to estimate the current baseline prevalence of conditions, and the potential impact of scaling-up a particular intervention (Modell et al. 2017). For example, improved diagnosis and care can extend the survival of children with incurable disorders, leading to an increasing number requiring ongoing care year-on-year. Therefore, countries at all levels of development need to assess their present situation with respect to congenital disorders, and the short- and long-term effects of implementing available interventions on patient numbers and service needs.

Information on current access to packages of care and interventions form an important part of estimation of the overall burden of congenital disorders, and the likely potential impact of investment of resources to scale-up these interventions. Currently, many countries have limited observational data on coverage of care. The methods described in this paper can provide estimates of access to services for countries without data, thus allowing burden estimation of congenital disorders to be undertaken for the purposes of policy and programme planning. However, these estimates rely on numerous assumptions as detailed above. Looking forward, substituting local data on the coverage of access to different interventions as it becomes available will substantially strengthen the MGDb burden estimates.

## Electronic supplementary material

ESM 1 (DOCX 115 kb)
