# Supplementary material for: Methods to estimate access to care and the effect of interventions on the outcomes of congenital disorders
Source: J Community Genet. 2018 Mar 17;9(4):363–76. doi: 10.1007/s12687-018-0359-3 (PMC6167260; doi:10.1007/s12687-018-0359-3)
Supplement: Supplementary file 1 — (DOCX 115 kb) [file 12687_2018_359_MOESM1_ESM.docx]

# Methods to estimate access to care and the effect of interventions on the outcomes of congenital disorders – online resource

Authors: Hannah Blencowe, Sowmiya Moorthie, Matthew Darlison, S Gibbons, Bernadette Modell, Congenital Disorders Expert Group

Congenital Disorders Expert Group: AH Bittles, H Blencowe, A Christianson, S Cousens, M Darlison, S Gibbons, H Hamamy, B Khoshnood, CP Howson, J E Lawn, P Mastroiacovo, B Modell, S Moorthie, JK Morris, PA Mossey, AJ Neville, M Petrou, S Povey, J Rankin, L Schuler-Faccini, C Wren, KA Yunis

Contents

[Estimating access to services 2](#_Toc482104337)

[Figure i: Correlation of other potential proxy indicators for access to care with infant mortality rate. 2](#_Toc482104338)

[Table i: Correlation of other possible demographic indicators with NNMR and IMR, 2010-2015 2](#_Toc482104339)

[Figure ii: Relation between neonatal and infant mortality, 1990 3](#_Toc482104340)

[Figure iii: Proportion of the population with access to services for 191 countries in 2010 3](#_Toc482104341)

[Methods used to fit a curve to the estimated access to using IMR groups: 4](#_Toc482104342)

[Methods used to calculate consanguinity-associated IMR: 4](#_Toc482104343)

[Methods used to calculate consanguinity-adjusted IMR: 5](#_Toc482104344)

[Table ii: Effect of adjustments to IMR on estimates of access to care 6](#_Toc482104345)

[Interventions that impact on birth outcomes 9](#_Toc482104346)

[Table iii: Reported effects of folic acid flour fortification at the population level 9](#_Toc482104347)

[Table iv: Effect of mandatory folic acid fortification in the US on anencephaly and spina bifida 10](#_Toc482104348)

[Effect of type of antenatal screening policy on rates of TOP for affected pregnancies: 10](#_Toc482104349)

[Legal status of TOP for fetal impairment. 11](#_Toc482104350)

[Table v: Countries where TOP for fetal impairment is legal 11](#_Toc482104351)

[Table vi: Countries with illegal or unclear legal status of TOP for fetal impairment 12](#_Toc482104352)

[Table vii: Policies on prenatal diagnosis in 14 EUROCAT countries in 2009 13](#_Toc482104353)

[References: 16](#_Toc482104354)

# Estimating access to services

### Figure i: Correlation of other potential proxy indicators for access to care with infant mortality rate.

Data source WPP 2012 revision (UN Population Division)

### Table i: Correlation of other possible demographic indicators with NNMR and IMR, 2010-2015

| **Indicator** | **Coefficient of correlation** | |
| --- | --- | --- |
|  | with NNMR | with IMR |
| **NMR and IMR** | 0.95 | 0.95 |
| **under-5 MR** | 0.93 | 0.99 |
| **Av. life expectancy** | -0.88 | -0.94 |
| **% urbanisation** | -0.62 | -0.63 |

Data Source: WPP 2012 revision (UN Population Division)

### Figure ii: Relation between neonatal and infant mortality, 1990

coefficient of correlation = 0.93

Data sources: neonatal mortality (IGME 2015)(IGME) and infant mortality (WPP 2015 revision)(Division; ) in 1990

### Figure iii: Proportion of the population with access to services for 191 countries in 2010

Chart shows step-wise nature of method used by CHERG giving rise to discontinuities, particularly as, with time, countries move across boundaries (blue line) (Blencowe et al. 2013). The red line shows refined method using an equation to estimate the access to care for countries with NMR of 6 – 15/1000 as used in GBD2010 (Vos et al. 2012).

## Methods used to fit a curve to the estimated access to using IMR groups:

The smooth function fitted to the infant mortality rate data is based on the standard Beta family of distributions, which can be used to provide a range of curves according to two shape parameters *a* and *b*. The continuous proportion with access to services (*p*) is predicted from the infant mortality rate using:

$\begin{matrix} p=1-I\left(\tilde{x};a,b\right)\text{ if }\tilde{x}\geq0 \\ p=1\text{ if }\tilde{x}<0 \end{matrix}$ (1)

Where $\tilde{x}=\ln\left(imr-c\right)/\ln\left(1000\right)$

In equation (1) *imr* is the infant mortality rate in deaths per 1,000 live births, and *c* is an offset parameter specifying the number of births below which it is assumed that the proportion with access to services is 1 (i.e. 100%).

The function $I_{\tilde{x}}\left(\tilde{x};a,b\right)$ is the regularised Beta function, which is defined as $I\left(\tilde{x};a,b\right)=\left\{\int_{0}^{\tilde{x}} u^{a-1}\left(1-u\right)^{b-1}du\right\}\frac{\left(a-1\right)!\left(b-1\right)!}{\left(a+b-1\right)!}$

The parameters a, b and c are estimated empirically, by visual fitting of the smooth curve to the stepped curve, yielding: *a* = 2.5, *b* = 5.5, *c* = 10.

The term ln(1000) in the denominator of the last line of equation (1) is a scaling parameter which ensures that the variable $\tilde{x}$ varies between 0 (no deaths) and a maximum of 1 (1000 deaths). The regularised Beta function is only defined over the range [0-1].

Equation (1) is implemented in Microsoft Excel, using the BETA.DIST function:

**Proportion with access = (1-BETADIST(LN(IMR-10),2.5,5.5,0,LN(1000)))**

## Methods used to calculate consanguinity-associated IMR:

Consanguinity-associated IMR (cIMR) is calculated as:

$$cIMR=(\left( F*100*a*\left( 1-b \right) \right)+(F*100*c*b)$$

Where:

F= local coefficients of consanguinity (Bittles 2001)

a= mortality from consanguinity-associated disorders in the absence of care. (2.08 per 1,000 per 0.01F calculated from (Bittles and Black 2010; Bittles and Neel 1994))

b= proportion with access to care (calculated from UN WPP country infant mortality estimates using methods as described above)

c= mortality from consanguinity-associated disorders with optimal care (1.44 per 1,000 per 0.01F calculated from (Bundey and Alam 1993))

## Methods used to calculate consanguinity-adjusted IMR:

Iteration 1:

Consanguinity_adjusted IMR1= IMR – cIMR1

Iteration 2:

Consanguinity_adjusted IMR2= IMR – cIMR2

Where

$$cIMR2=(\left( F*100*a*\left( 1-b2 \right) \right)+(F*100*c*b2)$$

F= local coefficients of consanguinity (Bittles 2001)

a= mortality from consanguinity-associated disorders in the absence of care. (2.08 per 1,000 per 0.01F calculated from (Bittles and Black 2010; Bittles and Neel 1994))

b2= proportion with access to care (calculated from UN WPP country infant mortality estimates using methods as described above using Consanguinity_adjusted IMR1 in calculation to estimate access to care)

c= mortality from consanguinity-associated disorders with optimal care (1.44 per 1,000 per 0.01F calculated from (Bundey and Alam 1993))

Iteration 3:

Consanguinity_adjusted IMR3= IMR – cIMR3

Where

$$cIMR3=(\left( F*100*a*\left( 1-b3 \right) \right)+(F*100*c*b3)$$

F= local coefficients of consanguinity (Bittles 2001)

a= mortality from consanguinity-associated disorders in the absence of care. (2.08 per 1,000 per 0.01F calculated from (Bittles and Black 2010; Bittles and Neel 1994))

b3= proportion with access to care (calculated from UN WPP country infant mortality estimates using methods as described above using Consanguinity_adjusted IMR1 in calculation to estimate access to care)

c= mortality from consanguinity-associated disorders with optimal care (1.44 per 1,000 per 0.01F calculated from (Bundey and Alam 1993))

### Table ii: Effect of adjustments to IMR on estimates of access to care

| **WHO region or sub-region** | **Births, 1,000s** | **IMR (WPP)** | **Contribution of** | | **% with access, based on** | | **Effect of adjustment** |
| --- | --- | --- | --- | --- | --- | --- | --- |
|  |  |  | Consanguinity-related IMR | HIV-related IMR | WPP IMR | IMR adj consang & AIDS | % increase in access |
| **Algeria, W Sahara** | 960 | 30.4 | 2.11 | 0.00 | 21 | 24 | 12.1 |
| **Sub-Saharan Africa** | 31,399 | 64.8 | 1.47 | 1.06 | 7 | 7 | 4.7 |
| **Southern Africa** | 1,871 | 42.1 | 1.48 | 2.50 | 13 | 15 | 6.4 |
| **AFR Total** | 34,230 | 62.6 | 1.49 | 1.11 | 7.7 | 8.4 | 5.5 |
| **North America** | 4,365 | 5.9 | 0.06 | 0.00 | 100 | 100 | 0.0 |
| **Caribbean** | 791 | 26.9 | 0.20 | 0.18 | 44 | 44 | 1.1 |
| **Central America** | 4,710 | 18.5 | 0.08 | 0.01 | 49 | 50 | 0.6 |
| **South America** | 4,414 | 21.7 | 0.33 | 0.01 | 39 | 40 | 2.7 |
| **S America HI** | 1,039 | 12.2 | 0.02 | 0.01 | 82 | 82 | 0.1 |
| **AMR Total** | 15,319 | 15.8 | 0.15 | 0.01 | 63 | 63 | 0.7 |
| **Gulf states** | 913 | 12.7 | 2.64 | 0.01 | 75 | 94 | 24.9 |
| **N Africa /Middle East** | 9,105 | 28.5 | 3.08 | 0.02 | 36 | 43 | 20.9 |
| **Pakistan, Afghanistan** | 6,419 | 70.1 | 6.72 | 0.01 | 5 | 6 | 21.4 |
| **Somalia, Dj, S Sudan** | 887 | 78.0 | 2.15 | 0.68 | 4 | 4 | 5.9 |
| **EMR Total** | 17,323 | 45.6 | 4.35 | 0.05 | 25 | 30 | 21.5 |
| **Western Europe** | 4,424 | 3.3 | 0.08 | 0.00 | 100 | 100 | 0.0 |
| **Central Europe** | 1,173 | 6.3 | 0.02 | 0.00 | 99 | 99 | 0.0 |
| **Eastern Europe** | 2,530 | 8.2 | 0.01 | 0.01 | 100 | 100 | 0.0 |
| **Turkey** | 1,304 | 12.6 | 1.05 | 0.00 | 87 | 98 | 12.3 |
| **Central Asia** | 1,865 | 33.4 | 2.19 | 0.01 | 31 | 33 | 6.8 |
| **EUR Total** | 11,296 | 10.7 | 0.52 | 0.01 | 87 | 88 | 1.8 |
| **South Asia** | 29,722 | 40.3 | 1.36 | 0.03 | 14 | 14 | 5.7 |
| **Southeast Asia** | 7,224 | 25.7 | 0.40 | 0.04 | 37 | 38 | 1.6 |
| **N Korea** | 358 | 22.0 | 0.00 | 0.01 | 36 | 36 | 0.0 |
| **SEAR Total** | 37,304 | 37.3 | 1.16 | 0.03 | 18 | 19 | 4.0 |
| **East Asia** | 16,938 | 11.7 | 0.11 | 0.01 | 96 | 98 | 1.0 |
| **Southeast Asia** | 4,941 | 21.6 | 0.16 | 0.02 | 41 | 42 | 0.8 |
| **Asia Pacific HI** | 1,838 | 2.6 | 0.07 | 0.00 | 100 | 100 | 0.0 |
| **Oceania** | 279 | 41.9 | 1.14 | 0.11 | 19 | 20 | 7.6 |
| **Australasia** | 372 | 4.0 | 0.02 | 0.00 | 100 | 100 | 0.0 |
| **WPR Total** | 24,368 | 13.3 | 0.13 | 0.01 | 85 | 86 | 0.9 |
| **World** | 139,840 | 35.8 | 1.30 | 0.29 | 39 | 40 |  |

#

# Interventions that impact on birth outcomes

### Table iii: Reported effects of folic acid flour fortification at the population level

|  |  | **NTD births /1,000** | |  |
| --- | --- | --- | --- | --- |
| **Country** | Folic acid ppm | Pre-fortification | Post-fortification | % decrease in birth prevalence |
| **Saudi Arabia** | 1.5 | 1.9 | 0.76 | 60 |
| **Brazil** | 1.5 | 2.63 | 2.11 | 19.8 |
| **Iran** | 1.5 | 3.16 | 2.19 | 30.7 |
| **Oman** | 1.5 | 3.9 | 1.22 | 68.7 |
| **South Africa** | 1.5 | 1.34 | 0.91 | 32.1 |
| **Canada** | 1.5 | 1.38 | 0.72 | 47.8 |
| **USA** | 1.54 | 1.08 | 0.69 | 35.8 |
| **Costa Rica** | 1.8 | 1.1 | 0.41 | 62.7 |
| **Chile** | 2.2 | 1.65 | 0.83 | 49.7 |
| **Argentina** | 2.2 | 2.13 | 1.03 | 51.6 |

Data source: Zimmerman 2010 (Zimmerman S 2010)

Footnote: Evidence suggests that folic acid supplementation or voluntary fortification has little impact, and we have therefore assumed no effect of these interventions, however this may be an under estimate as EUROCAT surveillance data shows an average birth prevalence of NTDs since 1980 of 0.88/1000 in Western Europe. This figure is only 6% higher than the 0.77/1,000 USA baseline for non-folic-acid-preventable neural tube defects, and may represent the minimum achievable with a high quality diet combined with voluntary folic acid or folate supplementation.

### Table iv: Effect of mandatory folic acid fortification in the US on anencephaly and spina bifida

|  | Pre-fortification | Post-fortification |
| --- | --- | --- |
| Disorder group | 1995–6 | 1999–2011 |
| Anencephaly | 0.42 | 0.29 |
| Spina bifida | 0.65 | 0.40 |
| Anencephaly & spina bifida | 1.07 | 0.69 |
| Estimated encephalocoele^a^ | 0.12 | 0.079 |
| Estimated total NTD | 1.19 | 0.77 |

^a^ calculated assuming prevalence of encephalocoele to be 11.5% of that of anencephaly based on EUROCAT average data

Data source: (Williams et al. 2015)

## Effect of type of antenatal screening policy on rates of TOP for affected pregnancies:

Linking registry data from EUROCAT with reported policy indicates that in European countries where TOP is legal restricted screening policy results in 50% lower rates of TOP for Down syndrome and spina bifida compared to countries with universal screening. Termination rates for anencephaly, other trisomies and Turner syndrome (all also detectable on routine ultrasound scanning and not requiring laboratory biomarkers or detailed anomaly scanning) are less affected (Boyd et al. 2008). In MGDb we have assumed 50% lower rates of TOP for Down syndrome and spina bifida for countries with restrictive policies of screening compared to countries with universal screening.

## Legal status of TOP for fetal impairment.

### Table v: Countries where TOP for fetal impairment is legal

| Albania | Czech Republic | Liberia | Singapore |
| --- | --- | --- | --- |
| Armenia | Democratic People's Republic of Korea | Lithuania | Slovakia |
| Australia | Denmark | Luxembourg | Slovenia |
| Austria | Eritrea | Mexico | South Africa |
| Azerbaijan | Estonia | Monaco | Spain |
| Bahamas | Ethiopia | Mongolia | Sudan |
| Bahrain | Fiji | Montenegro | Swaziland |
| Barbados | Finland | Namibia | Sweden |
| Belarus | France | Nepal | Switzerland |
| Belgium | Georgia | Netherlands | Tajikistan |
| Belize | Germany | New Zealand | Thailand |
| Benin | Ghana | Niger | The Former Yugoslav Rep. of Macedonia |
| Bosnia and Herzegovina | Greece | Norway | Togo |
| Botswana | Guinea | Oman | Tunisia |
| Bulgaria | Guyana | Panama | Turkey |
| Burkina Faso | Hungary | Poland | Turkmenistan |
| Cambodia | Iceland | Portugal | Ukraine |
| Canada | India | Qatar | United Kingdom |
| Cape Verde | Israel | Republic of Korea | United States of America |
| Chad | Italy | Republic of Moldova | Uzbekistan |
| China | Jordan | Romania | Viet Nam |
| Colombia | Kazakhstan | Russian Federation | Zambia |
| Croatia | Kuwait | Saint Vincent and the Grenadines | Zimbabwe |
| Cuba | Kyrgyzstan | Serbia |  |
| Cyprus | Latvia | Seychelles | |

Data Source: UN Population Division (UN Population Division 2013)

### Table vi: Countries with illegal or unclear legal status of TOP for fetal impairment

7 countries with births >1000 per year not included in WHO 2013 abortion policy as having legal TOP for fetal impairment, but with evidence to suggest widespread practice of offering option of TOP for fetal impairment when diagnosed prenatally

| Country | Evidence source |
| --- | --- |
| Iran (Islamic Republic of) | 1998 Fatwa issued by Ayatullah Sayyid Ali Khamenei allowing TOP for Shia Muslims for pregnancies effected by genetic abnormalities(Akhlaghpoor 2006)  2005, the Iranian Parliament and Council of Guardians approved a new bill allowing TOP in cases of non-viable fetus or disorders that would make it hard for the mother to look after the child(Mehryar et al. 2007) |
| Iraq | Evidence of widespread practice eg Al-Allawi et al (Al-Allawi et al. 2015) |
| Japan | Chapter XXIX of the Penal Code of Japan makes abortion de jure illegal in the country, however, the Maternal Health Protection Law allows approved doctors to practice abortion with the consent of the mother and her spouse, if the pregnancy has resulted from rape, or if the continuation of the pregnancy may severely endanger the maternal health because of physical reasons or economic reasons. TOP for severe malformations is widely available (Kitai et al. 1994; Nagase et al. 2011). |
| Pakistan | 1995 Fatwa Mohamed Taqi Ohmani, Jamia Dar u-Ulaam, Karachi allowing TOP for serious disorders(Ahmed et al. 2000; Jafri et al. 2012) |
| Egypt | Evidence of widespread practice eg Shalaby et al (Shalaby et al. 2012) |
| Haiti | Evidence of widespread practice of illegal TOP and increasing access to ultrasound eg Huber et al (Huber 2015) |
| Brazil | TOP for anencephaly and non-viable fetuses legalised by the supreme court in 2012 (Westphal et al. 2016) |

In addition of relevance to Islamic nations the Islamic Jurisprudence Council of the Islamic World League released in 1990 a religious advice (fatwa) allowing abortion in the presence of severe fetal malformations not amenable to treatment or leading to poor quality of life of patients and their relatives.

NOTE REGARDING TOP ASSUMPTIONS

A limitation of the approach used is that in many countries there are gaps between legal status, official policy and clinical practice, This is likely in mane settings to underestimate the number of TOPs, and hence will effectively overestimate the number of affected births and congenital associated mortality and disability. An example is Brazil, where whilst very recent legislation has legalised TOP for anencephaly and non-viable foetuses, TOP is otherwise illegal. However, availability of prenatal diagnosis and practice of TOP is widespread, with an estimate 1 in 4 pregnancies ending in TOP (Victora et al. 2011).

### Table vii: Policies on prenatal diagnosis in 14 EUROCAT countries in 2009

Data Source: Eurocat special report. Prenatal screening policies in Europe. 2010 (European Surveillance of Congenital Anomalies (EUROCAT) 2010)

| **Expectation for detection** | **High** | **Very high** | **High** | **35 plus only** | **Very high** | **High** | **Very high** | **35 plus only** |
| --- | --- | --- | --- | --- | --- | --- | --- | --- |
| **Country** | Austria | Belgium | Croatia | Denmark 1994-2004 | Denmark 2004 on | Finland | France | Germany |
| **Screening for Down syndrome** | No general policy. But inform women of prenatal tests, advice and risks. Public insurance pays for CVS and amnio if ≥35 at EDD and combined test & USS if >36 at conception | Universal nuchal translucency @ 11-13 wk & 1ts trim serum screen | No official guidelines. | Women 35 plus & those at increased risk | Universal 1st trim serum screen & US scan | Opt-in 1st trim serum screen & scan offered to all | Law from 1997: universal offer serum screen at 14-16 weeks, & nuchal translucency at 11-13 weeks | Advice that all M 35 plus have amnio. Voluntary triple test is charged. Recently nuchal translucency. |
|  |  |  | USS offered to all +/- biochemical tests |  |  | USS morphological screen at 18 - 22 wks |  |  |
| **Indications for prenatal cytogenetic diagnosis** | Mat age 35  Or FHX - genetic disorders in siblings, parents, relative or USS abn or abn serum screen or exp to teratogens/ high dose radiation or extreme anxiety | Mat age 36 plus. 1st trim serum screen risk, 1:250 or more  Sibling or parents with known chromosomal abn.  FHx DNA abn or metabolic disorder  USS abn suggestive of chromosomal abn | No official indications, but widely used:  Mat age 35 plus. Sibling or parents with known chromosomal abn. Exposure to radiation/ chemotherapy | Mat age 35 plus, 2nd trim serum screen risk 1:400 or more, US major malfn, other increased risk | 1st trim serum screen risk, (or any other risk) 1:250 or more, US major malfn | Mat age 40 plus  High risk from prenatal serum screen  Major abn on USS  previous affected child or parents known carriers | To 1988: women 38 or over. Since 1997, when screening indicates risk >1/250, or US abnormalities plus scan markers. All women 38 plus. | Woman 35 plus. Indication of increased risk (including blood group incompatibility) |
| **US screening for structural anomalies** | Voluntary at 20wk | Routine anomalies USS 2^nd^ trimester. | No official recommendation. | Any indication of increased risk | Universal offer at around 18 weeks | Universal offer at 18 – 22 weeks or after 24 weeks | 3 routine scans for structural abns: 12 wk, 22 wk including cardiac exam, 32 weeks | 3 routine scans: 9-12 wk, 19-22 wk, 29-32 wk |
|  |  | Specialised USS if abn, or RF teratogens, DM | Usually 3 – including around 18 – 24 weeks |  |  |  |  |  |
| **TOP for fetal anomaly** | Choice to 24 wks if severe abn (not isolated clefts). After 24 wks only non-viable cases or late diagnosis of very severe abn (not trisomy 21) | Choice to 12 weeks. Medical cert required if later. | TOP legal 1978  After 10 wks need medical panel consent. Upper limit 24 weeks | Choice to 12 weeks. Medical cert required if later. Legal after 24 weeks for v severe or lethal abn. | Choice to 12 weeks. Medical cert required if later. Legal after 24 weeks for v severe or lethal abn. | TOP legal up to 24 weeks | TOP permitted for serious abn no age limit. Review by committee. | TOP legal for risk of severe physical and mental risk to mat health, at any stage. |

Table Vii Continued…

| **Expectation for detection** | **None** | **35 plus only** | **38 plus only** | **Lowish** | **High** | **Very high** | **Very high** |
| --- | --- | --- | --- | --- | --- | --- | --- |
| **Country** | Ireland | Netherlands | Norway | Portugal | Sweden | Switzerland | UK |
| **Screening for Down syndrome** | No policy. Nuchal translucency on request | Policy = nuchal translucency 11-13 wk, triple test around 15 wk. Free only for women 36 wk plus. Younger women informed of test, must pay 60-80 EUROS | Woman 38 plus or at increased risk | 38/54 obstetric units have PND centres 2005. only 13 official recognition. Biochemical screening widespread especially in private labs. | No general policy. Nuchal translucency @ 11-13 wk routine | Universal offer. 1st or 2nd trim serum tests, US. Very complete. | universal offer |
|  |  |  |  |  |  |  |  |
| **Indications for prenatal cytogenetic diagnosis** | Available on individual request | Woman 36 plus, screening risk 1/250 plus, other increased risk | Woman 38 plus or at increased risk | Woman 35 plus (if resources exist), other increased risk. | Woman 35 plus or increased risk, or worried mother. Around 50% uptake in 2005. | Women 35 plus, 1st trim screen risk >1/300, 2nd trim screen risk > 1/380. US abn, other increased risk. Must pay for PND for parental anxiety. | Mother 35 plus, high risk screen result (>1/250), other indication of increased risk. |
|  |  |  |  |  |  |  |  |
|  |  |  |  |  |  |  |  |
|  |  |  |  |  |  |  |  |
|  |  |  |  |  |  |  |  |
|  |  |  |  |  |  |  |  |
| **US screening for structural anomalies** | Scan at around 18 weeks includes screening for structural anomalies | NO POLICY. women have scan at first visit: not aimed at detecting congenital defects | Woman 38 plus or at increased risk | Recommended 3 routine scans. | 1 routine scan at 16-17 wk including cardiac. | 2 scans, 10-12 wk & 20-23 wk. | Fetal anomaly scan at 18-20 weeks. (96% plus in England & Wales, around 60% in Scotland) |
|  |  |  |  |  |  |  |  |
| **TOP for fetal anomaly** | Not legal | Legal to 24 weeks. After 24 weeks only for severe cases, committee. | Regional commission. After 18 weeks needs very strong reason | Before 1997 TOP permitted to 22 wk. From 1997, TOP permitted to 24 weeks. No upper limit if lethal condition. Local, regional, national commissions. | TOP to 18 wk no problem. 18 weeks plus, national multidisc committee. TOP v rare after 22 weeks | TOP to 24th wk, though no legal limit. | TOP legal to 24 weeks, no limit for serious handicap. |
|  |  |  |  |  |  |  |  |
|  |  |  |  |  |  |  |  |
| **Comment** |  | Very limited policies. Reimbursed only if indication of increased risk. Clearly, reservations | Clearly strong reservations |  |  |  | N Ireland: no official policy |

Abbreviations used in table: wk=weeks; amnio=amniocentesis; abn=abnormality; malfm=malformation; FHx=family history; TOP=Termination of pregnancy

# References:

Ahmed S et al. (2000) Prenatal diagnosis of beta-thalassaemia in Pakistan: experience in a Muslim country Prenat Diagn 20:378-383

Akhlaghpoor S (2006) Chorionic villus sampling for beta-thalassemia: the first report of experience in Iran Prenat Diagn 26:1131-1136 doi:10.1002/pd.1572

Al-Allawi NA et al. (2015) Premarital screening for hemoglobinopathies: experience of a single center in Kurdistan, Iraq Public health genomics 18:97-103 doi:10.1159/000368960

Bittles A (2001) Consanguinity and its relevance to clinical genetics Clin Genet 60:89-98

Bittles AH, Black ML (2010) The impact of consanguinity on neonatal and infant health Early Hum Dev 86:737-741 doi:10.1016/j.earlhumdev.2010.08.003

Bittles AH, Neel JV (1994) The costs of human inbreeding and their implications for variations at the DNA level Nature genetics 8:117-121 doi:10.1038/ng1094-117

Blencowe H et al. (2013) Estimates of neonatal morbidities and disabilities at regional and global levels for 2010: introduction, methods overview, and relevant findings from the Global Burden of Disease study Pediatr Res 74 Suppl 1:4-16 doi:10.1038/pr.2013.203

Boyd PA, Devigan C, Khoshnood B, Loane M, Garne E, Dolk H (2008) Survey of prenatal screening policies in Europe for structural malformations and chromosome anomalies, and their impact on detection and termination rates for neural tube defects and Down's syndrome Bjog 115:689-696 doi:10.1111/j.1471-0528.2008.01700.x

Bundey S, Alam H (1993) A five-year prospective study of the health of children in different ethnic groups, with particular reference to the effect of inbreeding European journal of human genetics : EJHG 1:206-219

Division; UNP World Population Prospects: the 2015 Revision <http://esaunorg/wpp/indexhtm> 2015

European Surveillance of Congenital Anomalies (EUROCAT) (2010) Eurocat special report. Prenatal screening policies in Europe. 2010. from <http://wwweurocat-networkeu/prenatalscreeeningand%20diagnosis/>

Huber B (2015) Haiti's push for safe motherhood Lancet 386:641-642 doi:10.1016/s0140-6736(15)61490-8

IGME Levels and trends in child mortality. UN Inter-agency Group for Child Mortality Estimation childmortalityorg

Jafri H, Ahmed S, Ahmed M, Hewison J, Raashid Y, Sheridan E (2012) Islam and termination of pregnancy for genetic conditions in Pakistan: implications for Pakistani health care providers Prenat Diagn 32:1218-1220 doi:10.1002/pd.3987

Kitai H, Watanabe H, Sayama M, Kanemune M, Nishiyama M, Nishino R, Itoh K (1994) The acceptability of prenatal diagnosis in Japan International journal of technology assessment in health care 10:436-446

Mehryar AH, Ahmad-Nia S, Kazemipour S (2007) Reproductive health in Iran: pragmatic achievements, unmet needs, and ethical challenges in a theocratic system Studies in family planning 38:352-361

Nagase H, Ishikawa H, Nishikawa T, Kurosawa K, Itani Y, Yamanaka M (2011) Prenatal management of the fetus with lethal malformation: from a study of oligohydramnios sequence Fetal and pediatric pathology 30:145-149 doi:10.3109/15513815.2010.547552

Shalaby HA, Elhady RA, Gamal AM, Badry AA (2012) Prenatal diagnosis in low resource setting: is it acceptable? Journal of obstetrics and gynaecology of India 62:515-519 doi:10.1007/s13224-012-0185-1

UN Population Division World Population Prospects: the 2012 Revision <http://esa.un.org/wpp/>

UN Population Division (2013) World Abortion Policies

Victora CG, Aquino EM, do Carmo Leal M, Monteiro CA, Barros FC, Szwarcwald CL (2011) Maternal and child health in Brazil: progress and challenges Lancet 377:1863-1876 doi:10.1016/s0140-6736(11)60138-4

Vos T et al. (2012) Years lived with disability (YLDs) for 1160 sequelae of 289 diseases and injuries 1990-2010: a systematic analysis for the Global Burden of Disease Study 2010 Lancet 380:2163-2196 doi:10.1016/s0140-6736(12)61729-2

Westphal F, Araujo Junior E, Fustinoni SM, Abrahao AR (2016) Maternal risks and predictor factors for the termination of pregnancy in fetuses with severe congenital anomaly: experience from a single reference center in Brazil J Matern Fetal Neonatal Med 29:3762-3767 doi:10.3109/14767058.2016.1147557

Williams J et al. (2015) Updated estimates of neural tube defects prevented by mandatory folic Acid fortification - United States, 1995-2011 MMWR Morbidity and mortality weekly report 64:1-5

Zimmerman S (2010) Fifteen Years of Fortifying With Folic Acid: Birth Defects are Reduced and Healthcare Expenses are Averted 'Sight and Life' Magazine 25
